# Complete genome sequence of *Alteromonas stellipolaris* strain 6L2 isolated from surface seawater using an ethylene-α-olefin co-oligomer

**DOI:** 10.1128/mra.01285-25

**Published:** 2025-12-23

**Authors:** Ryo Iizuka, Takao Yoshida, Masaru Kawato, Sotaro Uemura

**Affiliations:** 1 Department of Biological Sciences, Graduate School of Science, The University of Tokyo, Tokyo, Japan; 2 Japan Agency for Marine-Earth Science and Technology (JAMSTEC) 13570, Yokosuka, Japan; 3 Core Research for Evolutional Science and Technology (CREST), Japan Science and Technology Agency, Tokyo, Japan; Montana State University, Bozeman, Montana, USA

**Keywords:** *Alteromonas stellipolaris*, polyolefin, plasmids

## Abstract

*Alteromonas stellipolaris* strain 6L2 was isolated from surface seawater via enrichment culture using an ethylene-α-olefin co-oligomer, a polyolefin model substrate, as sole carbon source. We report its complete genome sequence comprising a circular chromosome and a plasmid. The genome provides insights into bacterial colonization of polyolefin surfaces in marine environments.

## ANNOUNCEMENT

*Alteromonas stellipolaris* is a cold-tolerant marine bacterium with diverse metabolic capabilities (1–6), but its role in polyolefin degradation remains unclear. We report the complete genome of strain 6L2, isolated from surface seawater using enrichment culture with an ethylene-α-olefin co-oligomer (LUCANT HC-40), a polyolefin model. This also yielded *Pseudoalteromonas* sp. strain 6L1 (7).

Surface seawater was collected from Suruga Bay, Japan (34°44.93′N, 138°40.41′E) on 24 April 2024 during the R/V *Kaimei* research cruise (KM24-03 Leg 1) (7). The seawater was concentrated ∼1,000-fold (7) and inoculated into artificial seawater with 0.1% NH_4_Cl and 1% LUCANT HC-40 (Mitsui Chemicals) (7–9). After 74 days at 4°C, a small portion of the LUCANT material was streaked onto an agar plate with artificial seawater medium (10–12) and incubated for 11 days at 4°C to isolate strain 6L2, which was identified as *A. stellipolaris* by 16S rRNA gene analysis (11, 12). The strain was cultured aerobically in artificial seawater medium for 50 h at 10°C. Genomic DNA was extracted using Genomic-tip 20/G (Qiagen) and further purified with 1.0× ProNex Beads (Promega). It was sheared into 10–25 kbp fragments using Megaruptor 3 (Diagenode). Libraries prepared with SMRTbell prep kit 3.0 and SMRTbell gDNA amplification kit were treated with Revio Polymerase Kit (all from PacBio). Sequencing was performed using the Revio system (PacBio). SMRT Link (v13.1.0.221970) (PacBio) was used to trim adapter sequences from the sequencing reads. Circular consensus sequences with average base quality values below 20 were removed to obtain HiFi reads. These reads were filtered using lima (v2.12.0) (https://github.com/pacificbiosciences/barcoding) and pbmarkdup (v1.0.3) (https://github.com/PacificBiosciences/pbmarkdup) to remove the ultra-low PCR adapters and PCR duplicates, respectively. Filtlong (v0.2.1) (https://github.com/rrwick/Filtlong) was used to exclude short HiFi reads (<1,000 bp). The remaining reads were assembled, and circular contig overlapping ends were removed using Flye (v2.9.3-b1797) (13). Taxonomic classification and annotation were performed using DFAST (v1.6.0) (14–16). Genome completeness and contamination were evaluated using CheckM2 (v1.0.1) (17). Genomes were reoriented using seqkit (v2.10.1) (18), and genome alignment was performed using DiGAlign (v2.0) (19). Default settings were used for all software.

**Fig 1:**
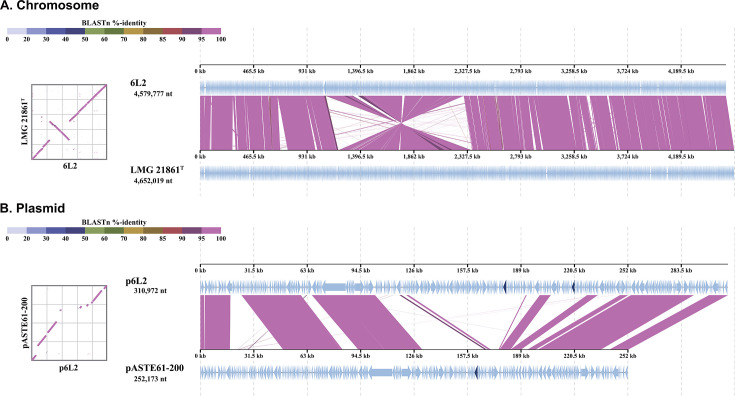
Genome alignment between *A. stellipolaris* strain 6L2 and LMG 21861^T^. (**A**) Chromosome. (**B**) Plasmid. (Left) Dot plot of synteny. (Right) Visualization of alignment. Colors indicate BLASTn percentage identity. Dark blue regions in the plasmid alignment indicate copper resistance system multicopper oxidase genes (two genes in the 6L2 plasmid [p6L2] and one gene in the LMG 21861^T^ plasmid [pASTE61-200]). The genomic sequence of LMG 21861^T^ was obtained from GenBank (chromosome, CP013926.1; pASTE61-200, CP013927.1). Chromosomes and plasmids were reoriented to start at the *dnaA* gene and the ParA family protein gene using Seqkit (v2.10.1) (18). Comparative analysis was performed using DiGAlign (v2.0) (19).

The 6L2 genome was assembled into a chromosome and a plasmid (Table 1). The closest relative was *A. stellipolaris* LMG 21861^T^ (GCA_001562115.1), with an average nucleotide identity of 98.73%. However, chromosome alignment revealed substantial rearrangements between the two strains (Fig. 1A). The plasmid differs substantially in size and structure from that of LMG 21861^T^ (Fig. 1B). The genome encodes exopolysaccharide-related proteins, suggesting biofilm formation on LUCANT surfaces. We identified two divergent genes encoding copper resistance system multicopper oxidases on the plasmid (Fig. 1B). Although their specific function in LUCANT degradation is unclear, these enzymes may contribute to extracellular polyolefin fragmentation, as suggested by their role in *Acinetobacter baumannii* (20). The plasmid localization of these genes implies horizontal transfer of polyolefin-degrading capabilities in marine environments. The genome lacks alkane hydroxylase genes, crucial for aerobic alkane degradation (21, 22). This suggests LUCANT utilization may involve novel mechanisms beyond conventional pathways. These findings enhance understanding of polyolefin colonization by marine bacteria.

**TABLE 1:** Sequencing statistics, genomic features, and accession numbers of strain 6L2

Parameter	Value for strain 6L2
Sequencing results	
Number of reads	39,474
Sum of length (bp)	248,867,583
Read N50 (bp)	7,011
SRA accession number	DRR786054
Assembly results	
Length (bp)	
Chromosome	4,579,777
Plasmid	310,972
GC content (%)	
Chromosome	43.6
Plasmid	42.4
Mean coverage (×)	50.9
Annotation results	
Number of protein-coding sequences	
Chromosome	3,874
Plasmid	303
Number of rRNAs	
Chromosome	15
Plasmid	0
Number of tRNAs	
Chromosome	62
Plasmid	0
Completeness check	
Completeness (%)	100
Contamination (%)	0.05
GenBank accession number	
Chromosome	AP044778.1
Plasmid	AP044779.1

## Data Availability

The genome sequence of strain 6L2 has been deposited in GenBank under accession numbers AP044778.1 and AP044779.1. Raw sequencing reads are available in SRA under accession number DRR786054. These are associated with BioProject PRJDB21836 and BioSample SAMD01693679.
